# Patient Machine Interface for the Control of Mechanical Ventilation Devices

**DOI:** 10.3390/brainsci3041554

**Published:** 2013-11-15

**Authors:** Rolando Grave de Peralta, Sara Gonzalez Andino, Stephen Perrig

**Affiliations:** 1 Electrical Neuroimaging Group, Albert Gos 18, Geneva 1206, Switzerland; E-Mail: sara.gonzalezandino@electrical-neuroimaging.ch; 2 Geneva Sleep Lab, Department of Neuropsychiatry, Geneva University Hospital (HUG), Geneva 1225, Switzerland; E-Mail: stephen.perrig@hcuge.ch; 3 Neural Microcircuitry Lab, École Polytechnique Fédérale de Lausanne (EPFL), Lausanne 1015, Switzerland

**Keywords:** patient machine interface, mechanical ventilation, neurodrive, EEG (electroencephalography), breathing, BCI (brain computer interfaces)

## Abstract

The potential of Brain Computer Interfaces (BCIs) to translate brain activity into commands to control external devices during mechanical ventilation (MV) remains largely unexplored. This is surprising since the amount of patients that might benefit from such assistance is considerably larger than the number of patients requiring BCI for motor control. Given the transient nature of MV (*i.e.*, used mainly over night or during acute clinical conditions), precluding the use of invasive methods, and inspired by current research on BCIs, we argue that scalp recorded EEG (electroencephalography) signals can provide a non-invasive direct communication pathway between the brain and the ventilator. In this paper we propose a Patient Ventilator Interface (PVI) to control a ventilator during variable conscious states (*i.e.*, wake, sleep, *etc.*). After a brief introduction on the neural control of breathing and the clinical conditions requiring the use of MV we discuss the conventional techniques used during MV. The schema of the PVI is presented followed by a description of the neural signals that can be used for the on-line control. To illustrate the full approach, we present data from a healthy subject, where the inspiration and expiration periods during voluntary breathing were discriminated with a 92% accuracy (10-fold cross-validation) from the scalp EEG data. The paper ends with a discussion on the advantages and obstacles that can be forecasted in this novel application of the concept of BCI.

## 1. Introduction

Breathing insures the exchange of oxygen and carbon dioxide between the air and the blood to maintain essential functions of the organs of the body on a moment by moment basis [1]. Breathing is subject to both voluntary and automatic control. Voluntary control adjusts breathing during daily activities such as speaking or eating and is thought to be regulated by cortical and subcortical centers involved in motor control as well as other areas involved in the specific acts being performed. Breathing continues during sleep or even during unconscious states due to the automatic control guaranteed by the coordinated action of networks of neurons in respiratory centers (RC) situated in the brainstem (pons) and the medulla [1]. The RC set the automatic breathing and send the impulses through the phrenic nerve to control the respiratory muscles (RM), including the diaphragm, that inflate and deflate chest wall and the lungs to cycle between the inspiration and expiration phases of breathing. The output of the respiratory centers that activates these vital skeletal muscles is called the respiratory or neural drive (ND) [2].

When the mechanics of breathing is compromised or the neural drive is diminished mechanical ventilation is required. For instance, decreased levels of consciousness (e.g., anesthesia, sleep, *etc.*) often coincides with reduced respiratory drive that eventually leads to poor oxygenation and apnea [1]. Other clinical conditions [3], e.g., acute respiratory failure, coma, chronic obstructive pulmonary disease or neuromuscular disorders also require the use of mechanical ventilation (MV) to decrease the work of breathing in the patients. The need for mechanical ventilation is indeed considered the chief reason for admission of patients in intensive care units (ICU) [3].

The experience accumulated over these years has lead to the conclusion that one of the most important aspects to insure that MV fulfills its role is to guarantee a correct coordination between the patient’s respiratory effort and the ventilator [3], *i.e.*, the correct synchrony between the patient’s neural drive and the machine. Indeed, patient-ventilator asynchrony (or dysynchrony) creates substantial imposed loads which can lead also to muscle fatigue and discomfort, increasing the dependence of the patient from the ventilator. Consequently, improving the synchrony between the patient and the ventilator is becoming a major goal in MV.

In parallel with developments in the field of MV we have seen over last decades important progresses in the field of neural prosthetics and non-invasive Brain Computer Interfaces (BCI) [4,5,6]. Thus far the BCI field has mainly evolved into the direction of assisting, augmenting or repairing sensory-motor functions with particular emphasis in completely paralyzed patients [7]. However, the potential of BCIs to establish direct communication pathways between the brain and other external devices such as mechanical ventilators remains largely unexplored. This is indeed surprising as the amount of patients that might benefit from advances in the BCI technology to control mechanical ventilation devices is considerably larger than the number of patients requiring BCI for motor control.

In this paper we discuss the basic schema of a non-invasive BCI system aimed to establish a direct communication pathway between non-invasively recorded brain signals in patients and mechanical ventilators. We start by discussing the clinical conditions requiring the use of mechanical ventilation to describe the potential of such technology. After a brief description of some of the recent progress in the field of mechanical ventilation we discuss some issues on the neural control of breathing to extract conclusions on the type of neural signals that can be used for the on-line control of the device. The paper finishes with a brief discussion on the main obstacles that can be forecasted in this endeavor.

## 2. Mechanical Ventilation and Neural Signals Associated to Voluntary and Involuntary Breathing

### 2.1. Current Needs for Mechanical Ventilation

While artificial respiration techniques have a long history dating back to the 18th century (for an historical perspective see [1] chapter 1), mechanical ventilation became a widespread technique only after the Scandinavian polio epidemic in 1952 where bag ventilation reduced the mortality from 80% to 40%. The 1960s, sometimes called the era of respiratory intensive care [8], witnessed the advent of the first mechanical ventilators. Since then, several types of ventilators and modes of ventilations have been proposed [3,9,10,11,12].

**Figure 1:**
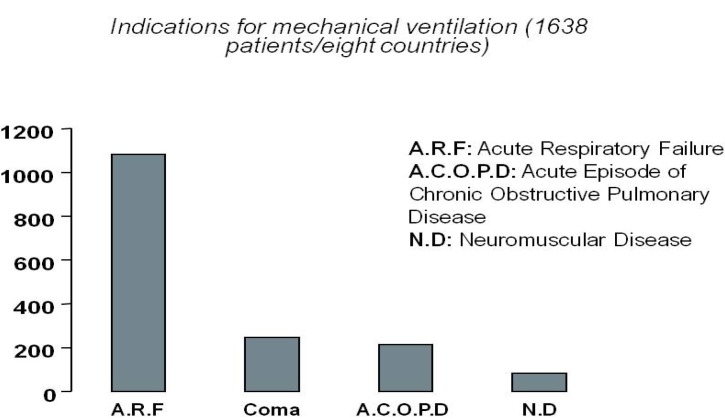
Main indications for mechanical ventilation in the intensive care unit (ICU). The causes leading to the need for mechanical ventilation are much more varied than the causes leading to motor paralysis. Therefore, the amount of patients that might benefit from a patient ventilator interface is considerably larger than the number of patients that might benefit from a motor oriented Brain Computer Interface.

Mechanical ventilation is required when there are clinical or para-clinical signs that the patient cannot maintain an airway or adequate oxygenation or ventilation [13]. Causes are multiple, ranging from chronic to acute diseases or emergency situations such as drowning or toxic effects to the CNS (Central Nervous System) caused by drugs. The leading indications for mechanical ventilation in the ICU are summarized in Figure 1 and were derived from a study of 1638 patients in eight countries [14]. The first group includes the acute respiratory distress syndrome, heart failure, pneumonia, sepsis, complications of surgery, and trauma (with each subgroup accounting for about 8% to 11% of the overall group). Neuromuscular diseases leading or not to complete paralysis can also impair the ability of respiratory muscles to impel air in/out the lungs. Examples of additional diseases requiring mechanical ventilation are: muscular dystrophies, motor neuron disease, including ALS, damage to the brain’s respiratory centers, polio, myasthenia gravis, myopathies affecting the respiratory muscles or even severe scoliosis.

Mechanical ventilation may also be required when the airway is obstructed, especially at night in sleep apnea. Sleep poses special requirements for mechanical ventilation and emphasizes the need for automatic control mechanisms. Indeed, even if a patient has the suitable means to voluntarily adjust the ventilator during the day, the ventilator needs to be switched to automatic control during the night to allow sleeping. Consequently, mechanical ventilation may be required only for a short period during stays at the ICU, only at night, or during limited daytime hours. Others patients require it chronically after their disease progresses.

The causes leading to the chronic or acute use of MV are much more varied than the causes leading to significant motor paralysis. Indeed, patients requiring a Brain Computer Interface to restore or augment motor function due to severe neuromuscular diseases (e.g., amyotrophic lateral sclerosis) or head or high spinal cord injuries (e.g., locked-in or quadriplegia) often require MV during the rest of their lives. Thus, BCI research might eventually contribute to improve the living standards of a population of patients considerably larger than the one targeted by conventional applications to sensory-motor function.

### 2.2. Current Advances and State of the Art

MV aims to avoid fatigue in patients while keeping the gas exchange vital for life. Typically, during MV, the patient inspiratory effort triggers a ventilator that pumps a mixture of air (Oxygen + other gases) through the central airways into the lungs inflating them and increasing the intraalveolar pressure. When the ventilator stops the central airway pressure decreases and air passively flows from the higher pressure lungs to the lower pressure central airways [15].

Patients with acute respiratory failure can reach inspiratory efforts as high as 4 to 6 times the normal value [3], increasing the work of the respiratory muscles and consequently increasing the risk of fatigue. While a careful selection of ventilator settings can reduce the patient efforts to its normal range it is not advisable to completely remove inspiratory effort as it might cause deconditioning and atrophy of respiratory muscles [3] compromising weaning from mechanical ventilation. In addition, the simple act of connecting a patient to a ventilator does not automatically decrease respiratory effort. A poor interplay between the patient and the ventilator can actually do the opposite and bring the patient to “fight” against the ventilator.

There is therefore a trade-off in MV where inspiratory efforts should be reduced but not completely eliminated. Although clear strategies and recommendations exist about ventilation parameters (e.g., flow, frequency, volume, pressure, *etc.*) the problem of insuring an optimal interplay or coordination or synchrony between the mechanical ventilation device and the subject respiratory efforts remains open. One key aspect of this interplay is the method used to trigger the ventilator which might influence both the patient-ventilator coupling as a whole (e.g., trigger asynchrony produced by ineffective triggering) as well as the cycle duration.

When the ventilator is not automatically activated it can be triggered by:

(1) Pressure trigger. The patient effort reduces the pressure till a cutoff value (sensitivity) that, when surpassed, activates the ventilator. The use of this technique is probably linked to the fact that pneumologists consider the mouth, the transdiaphragmatic and/or the esophageal pressures (P_es_) as closely related to the neural respiratory drive [16,17,18]. Nevertheless, existing literature (see [3] and references there in) confirms that up to one third of patient efforts can be unrewarded with this method.
(2) Flow trigger. The ventilator is activated when the flow, induced by the patient effort, surpass a cutoff value (sensitivity). Though considered more sensitive than (1), it might fail to avoid or eliminate patient-ventilator asynchrony [19].
(3) Electrical activity (electromyogram) of the diaphragm [10,20]. The ventilator is activated when the integrated electrical activity of the diaphragm (E_adi_) surpasses a threshold. The pressure and flow are also proportional to measured E_adi_.

In principle, these three methods can be implemented in a non-invasive way based on external measurements. However, due to the noisy character of external measurements, current practice often prefers the use of invasive esophageal recordings.

One of the more recent and successful approaches, called NAVA (Neurally Adjusted Ventilatory Assist [10]), requires an array of electromyogram sensors placed in the esophagus, at the level of the diaphragm, to detect inspiratory muscle activity (E_adi_) and set the pressure and flow of the ventilator. It has been considered as the ultimate patient-ventilator synchrony approach [12] and is expected to be [21] the solution to overcome ineffective triggering, expiratory asynchrony and flow asynchrony.

However, the neural drive generated at respiratory centers must travel along the nerves to activate the motor units of the diaphragm and induce a change in the pressure, flow or the electromyogram. Therefore, even the invasive measurements E_adi_ and P_es_ are just indirect and delayed reflects of the neural drive produced at respiratory centers of the brain. Furthermore, these invasive measurements provide scarce information (*i.e.*, feedback) about the effectiveness of the procedure as perceived by the patient. However, all this information is available to the neural centers responsible for the control of breathing and thus reflected in the electric activity of the brain. In our opinion, this electrical brain activity comprises all the information needed for the implementation and the assessment of a non-invasive patient ventilator interface with a high synchrony with the patient. In the next two sections we discuss the type of neural control signals that can be used for this purpose as well as the challenges behind this new alternative.

### 2.3. Neural Control Signals for a Patient Ventilator Interface (PVI)

**Figure 2:**
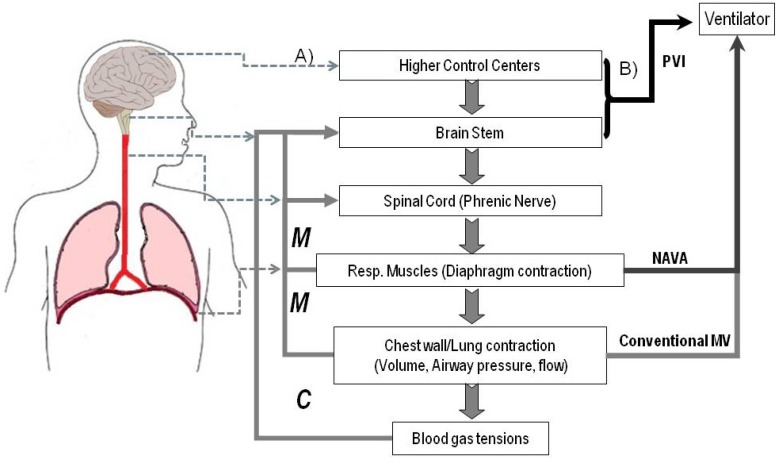
Basic schema of afferent and efferent processes in the control of breathing (*left*) and control signals that can be used to interface patients with ventilators (*right*). (**A**) During non-assisted (natural) breathing diverse cortical/subcortical structures and brainstem respiratory centers sent motor commands via the phrenic nerve to the respiratory muscles to insure the exchange of oxygen and carbon dioxide between the air and the blood. A moment by moment control loop is established by a variety of afferent signals sent back to the CNS/peripheral system by chemical (*C*) and mechanical (*M*) receptors; (**B**) In conventional ventilation transdiaphragmatic and/or esophageal pressures, or air flow are used to trigger the ventilator. In NAVA (Neurally Adjusted Ventilatory Assist), the electrical activity (electromyogram) of the diaphragm is used for control. In the Patient Ventilator Interface (PVI) we are proposing here, the control signals directly come from the brainstem (and eventually cortical) centers responsible for the respiratory rhythm and its automatic assessment (corollary/afferent responses).

Figure 2 depicts schematically the different control signals that are most commonly used in current clinical practice to trigger and control the ventilator and roughly introduce the schema of the PVI based on brain signals discussed next.

#### 2.3.1. Brainstem Signals Driving the Respiratory Rhythm

The more natural neural signals that can be used to control a patient ventilator interface are obviously those responsible for the generation of the respiratory rhythm. The great advantage of using these signals is that they convey continuous information about the patients’ requirements in all circumstances, e.g., during both conscious (awake) and unconscious (e.g., sleep, anesthesia) breathing, while accounting for the incoming afferent signals that reflect the state of the peripheral organs (e.g., muscles, lungs, *etc.*) involved in respiration.

The respiratory rhythm induced by the neural drive is generated by interacting populations of neurons distributed within the dorsal and ventral respiratory groups in the medulla. Additional nuclei in the pons—the pontine respiratory group—modulate and regulate this basic rhythm. The neurons of the ponto-medullary respiratory network drive two distinct pools of motoneurones: (1) motoneurons within the spinal cord innervating the diaphragm and the intercostals muscles; and (2) motoneurons within the nucleus ambiguous placed within the ventral respiratory group that coordinate pharyngeal, laryngeal and bronchial muscles to control airway resistance/airflow. These spinal and cranial motor activities have to be precisely coordinated to ensure efficient ventilation.

While brainstem centers provide the most natural control signal they are buried deep in the brain. This could hamper the detection of the signals from non-invasive EEG electrodes. There are however, two arguments supporting the idea that physiologically inspired signal processing techniques might help to overcome this limitation.

The first argument is the long experience accumulated in clinical neuroscience in the recording and analysis of acoustic brainstem potentials [22]. Indeed, the brainstem regions involved in breathing control is located “higher”, that is, closer to the skull, than the neuronal networks producing the brainstem auditory evoked potentials (BAEP). It is therefore surprising that little effort has been thus far placed on the problem of the estimation of the respiratory neural drive from non-invasive EEG measurements. Difficulties and workarounds on this approach are discussed later.

The second argument comes from the natural rhytmogenesis inherent to respiratory brainstem centers and which makes them suitable for the spectral analysis we are familiar with in sensory-motor BCI. In fact, one of the main issues in non-invasive BCI is the separation of activity coming from nearby centers from the activity that is targeted as control signal. Interestingly, and as happens with the neural discharges of many motor systems, it has been observed that high-frequency oscillations (HFO, 50–100 Hz in decerebrate cats and 100–150 Hz in rats) are ubiquitous in the discharges of motoneurons (phrenic, recurrent laryngeal, external intercostals) and of medullary neurons [23,24,25]. Moreover, the amplitude and frequency of HFO are greater when respiratory drive is increased suggesting that the spectral amplitude changes might provide information about the patient-ventilator synchrony. The analysis of coherence shows that the various discharges (population and unit) are significantly correlated at the HFO frequency. This indicates that the common rhythm arises in the brainstem and is transmitted further to cranial and spinal motoneurons [25]. We can therefore expect that the experience we have accumulated in the detection and analysis of HFO at the single trial level for sensory-motor BCI [26] could be extrapolated to the case of PVI now aimed to the detection of the neural drive at the brainstem level.

#### 2.3.2. Cortical Premotor Signals for a Posteriori Assessment

Inspiratory premotor potentials reflect the involvement of premotor cortical networks in the compensation of mechanical respiratory loading [27,28,29]. It has been shown that breathing against a mechanical load (e.g., an inspiratory resistance) evokes Bereitschaft or readiness potentials, which reflect the slowly increasing cortical excitability [30] related to the preparation of self-initiated movements [27,29]. Respiratory-related premotor potentials have been additionally described in relation with self-paced, voluntary sniff maneuvers [27] as well as during ventilator asynchrony in normal humans [31]. Premotor potentials could thus be markers of patient-ventilator asynchrony at the cortical level [31].

The cortical origin of premotor potentials facilitates the detection of this signal by scalp EEG electrodes and its potential use within a PVI. However, the absence of premotor potentials during effortless breathing or sleep precludes its use as a continuous control signal to trigger the ventilator according to the patient’s respiratory rhythm. The on-line detection of Bereitschaft or premotor potentials during mechanical ventilation can be then seen as a complement to brainstem signals within the control loop of a PVI as a marker of the patient-ventilator synchrony at a higher time scale. That is, we would probably need some unsuccessful breaths before having a premotor potential. Other types of control signals that can be better suited for such purposes are now discussed.

#### 2.3.3. Closing the PVI Loop in the Respiratory Cycle: Corollary/Afferent Responses as a Potential Source of Scalp EEG Feedback Related Potentials

In the more conventional applications of BCI to motor control, feedback is typically presented to the users to allow them assessing the performance of the system. When the response of the controller does not match user intentions error related potentials detected at the scalp surface can be used to correct inappropriate commands [32,33].

While in the case of the PVI there is not much sense in constantly giving visual, auditory or haptic feedback to the patients, it is however essential to monitor and assess the on-line performance (synchrony) of the patient-machine loop to introduce corrective measures (e.g., adjustment of ventilatory settings) when required. We believe that the best way to monitor such adjustment is to exploit the natural signals on which respiratory neural centers rely upon for similar purposes, *i.e.*, afferent signals from peripheral receptors and the closely linked corollary discharges.

As shown in Figure 2, the brainstem controller receives afferent information from a variety of chemical (*C* in Figure 2) and mechanical (*M*) sources. Some of these involve the relatively straightforward chemoreceptor signals that provide closed-loop information on the gas exchange functions of the lung. These signals arise mainly from the central and peripheral chemoreceptors that mediate the response to the levels of oxygen and carbon dioxide. There are also important receptors in the lung and the upper respiratory tract that provide afferent mechanical information to the respiratory centers. This information is used in normal respiration (e.g., vagal feedback of lung volume from pulmonary stretch receptors) as well as to initiate maneuvers such as sneezing and coughing that need to override the gas exchanging role of the respiratory system.

It is widely accepted that dyspnea (air hunger) may result when the need for ventilation (afferent neural signaling) is not being met by the physical breathing that is occurring (efferent neural signaling) [34]. This is a situation likely to occur when there is a patient machine asynchrony [29]. The feeling of dyspnea is thought to arise from central processing centers in the brain that compare the afferent and efferent signals and produce a mismatch response. While the neural origin and scalp morphology of mismatch signals is far from clear, it is not be surprising that responses to such a mismatch, in the case of breathing, resemble the ERP category that has been termed feedback-related potentials [35,36,37,38,39,40]. Indeed, multiple studies reviewed in [41] suggest that the generation (and suppression) of sense of respiratory work/effort and the generation (suppression) of “air hunger” are respectively due to neural copies (corollary discharges) of cortical respiratory or brainstem centers projecting to the forebrain. Investigating the scalp morphology or and topography of this speculated scalp EEG feedback related signals is then an interesting research line with potential use on the development of a PVI and the assessment of the patient ventilator synchrony.

Importantly, feedback related signals should exist during both automatic and voluntary breathing to shape the sense of respiratory effort. During automatic breathing the respiratory centers send the motor output to motor neurons and its exact (corollary) copy to sensory cortex. During voluntary breathing, the motor cortex sends a motor output directly to motor neurons and its exact copy to the sensory cortex [42]. Shedding light on the electrophysiological markers of these “respiratory motor command corollary discharges” might have implications beyond a simple PVI as the specific pathways and receptors for these signals have not yet been identified in humans.

#### 2.3.4. Illustrating the Proposed Approach: Discriminating the Inspiratory and Expiratory Periods during the Voluntary Control of Breathing in a Healthy Control

As in any other BCI, what is initially needed for the practical implementation of the proposed PVI is the continuous on-line extraction of the neural parameters that correlate with the respiratory rhythm. The key step is then to identify features that are reproducible markers of the dynamic of the respiratory rhythm. As is currently done in many other modalities of BCIs, this step requires counting with short pieces of EEG where the event we would like to discriminate and later recognize online (here, the neural command to the inspiratory muscles) is present and other windows where it is absent. Triggers to isolate these windows for the case of breathing can be added to the continuously recorded EEG from external devices that measure the onset of the air inflow into the mouth or alternatively from the electromyographic signals recorded on the chest and coming from the phrenic nerve. This approach is similar to the use of EMG signals to trigger the EEG linked to the readiness or Bereitschaft potential. This initial step is typically performed in healthy volunteers and/or patients with clear EMG signals or clearly detectable air inflow.

The presence of triggers serves to conveniently isolate the temporal EEG windows for feature selection in a binary classification problem. The binary problem to solve is to determine if a neural command for inspiration has been sent or not. The conventional approaches to feature selection [43] that serve to determine the best frequency bands and electrodes to create classifiers that optimally solve the on-line classification problem can be then applied.

To illustrate all the involved steps we collected data from a healthy control asked to produce self-paced voluntary inspiratory-expiratory loops while breathing through a face mask and a pneumotachograph. This allowed us to measure the airflow simultaneously with the EEG at locations C3 and C4 referenced to Cz at 256 Hz rate.

Our aim in this case was to evaluate the possibility to discriminate the inspiratory from the expiratory periods from the scalp recorded EEG. The flow signal coming from the pneumotachograph was segmented into the inspiration and expiration periods using a homemade based segmentation algorithm implemented in Matlab and comprising the following steps. (1) The airflow curve is smoothed to suppress the frequencies higher than 20 Hz; (2) Inspiratory-expiratory cycles are identified from the zero crossing of the smoothed airflow; and (3) Define the inspiratory (expiratory) onset as the zero preceding the positive (negative) part of each cycle.

**Figure 3:**
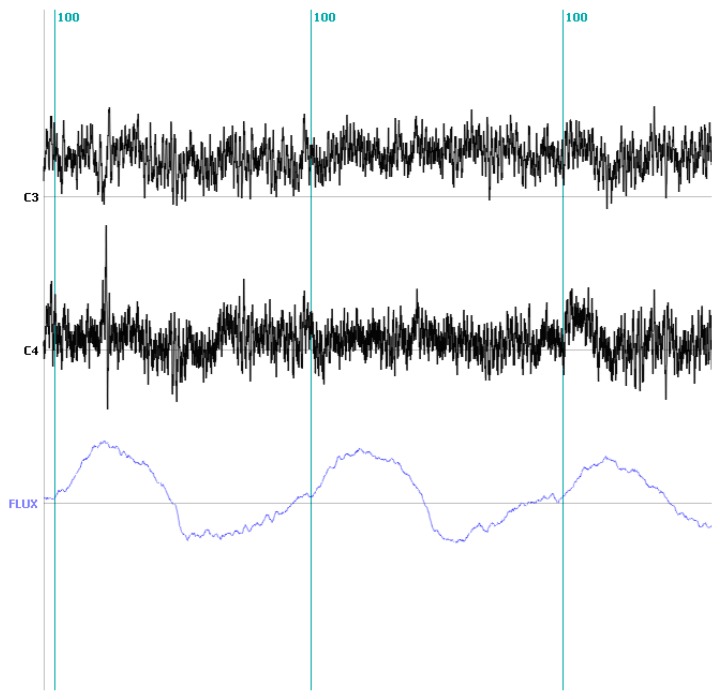
EEG traces measured on C3 and C4 (*black*) during inspiratory and expiratory effort in a healthy volunteer. The lower (*blue*) trace depicts the air flow simultaneously measured by a pneumotachograph. The vertical lines (*marker 100*) denotes inspiration onset as detected from the flow signal.

Around 240 EEG segments of one second duration were selected at the beginning of the inspiratory (120) and expiratory (120) part of the cycle from sensors C3 and C4 which are approximately placed over the somatosensory cortex from which voluntary control signals should arise. Examples of the raw EEG on C3 and C4 together with the pneumotachograph (flow) signal are given in Figure 3.

Features for classification were selected from the whole frequency band from zero to the Nyquist frequency (128 Hz) and were composed by the power spectrum of the different frequency components as obtained from simple Fourier analysis. To rank features according to their capabilities to distinguish the two classes we relied on the discriminative power (DP) as described in [43]. This measure provides an estimate of the rate of true positives that can be obtained classifying with each single feature given that the number of false positive is set to zero and can be therefore used to rank individual features, *i.e.*, power at each frequency and electrode, according to its individual capability to differentiate the two classes.

Using the ranking provided by the DP we selected the best 60 features and used a linear SVM to classify the data following a ten-fold cross-validation approach. That is, the data set was therefore divided into 10 subsets and each subset is used once as a test set while the complementary nine subsets are used as training set to select the features and compute the classifier parameters. Consequently, every data point (*i.e.*, each trial) is a member of the test set only once and a member of the training set nine times.

The correct classification rate averaged over the 10 folds reached 92% for this subject. While this is a fairly accurate result, it must be acknowledged that automatic breathing control might posit a more difficult challenge as discussed next.

### 2.4. Challenges and Workarounds in Developing a Patient Ventilator Interface (PVI)

The more difficult challenge for the practical implementation of the proposed PVI is the detection of the tiny signals generated at the brainstem respiratory centers which are volume conducted to the scalp surface. While there is ample experimental evidence that BAEPs can be recorded at the scalp, the reconstruction of the BAEP’s waveshape typically requires hundreds of repetitions. It must, however, be noted that the main goal in PVI is not to accurately reconstruct or estimate the waveshape as in conventional Event Related Potentials (ERP). The goal is rather to make decisions about the presence or absence of a neural drive on a short temporal window. This is a much simpler binary classification problem that has been successfully addressed by the BCI community. It has been indeed shown [43,44] that it is possible to detect the onset of a given mental state using very short time windows without resorting to the reconstruction of the time course of the target signal.

The example of the readiness or Bereitschaft potential [45], often used in some of the existing BCIs aimed to motor control [4,46,47], is illustrative of the differences between the characterization of the signal waveform and the detection of its presence or absence. According to the EEG literature, characterizing the ERP waveform linked to a readiness potential requires averaging over hundreds of trials [45]. However, its presence or absence and laterality can be readily identified from single sweeps, as repeatedly demonstrated in the BCI literature [7]. The problem in BCI is not that of signal extraction but that of signal identification which is certainly easier to tackle. This is simplified in the case of breathing as it corresponds to a binary decision: either the patient wants to (voluntarily or involuntarily) trigger the machine on the analysis window or not.

Note also that that the generator of the readiness potential at the Supplementary Motor areas is as far away from frontal scalp electrodes as the brainstem from electrodes placed on the neck. On this basis we cannot discard the possibility of detecting signals related to the voluntary and automatic control of breathing from scalp EEG and this issue deserves further investigation.

The rhythmic character of the neural drive and its spectral characterization within the very high frequency band might be the crucial fact for its fast detection and tracking in real time. Indeed, there is a distinction between the high frequency band (gamma band) typically defined between 30–80 Hz and the so-called epsilon oscillations where the respiratory rhythm seems to be inserted (above 100 Hz, termed here very high frequency oscillations). Gamma band activity appears as a ubiquitous marker of cortical activity. However, ECoG (electrocorticogram) as well as studies aimed to understand the relationship between spiking activity and LFP signals or EEG have shown that epsilon oscillations are very focal and circumscribed to areas where spiking activity is observed [48,49]. Moreover their timing and selectivity resembles that of spiking activity [50]. For a short discussion on the role of epsilon oscillations in BCI control and more references on their origins and selectivity see [26].

Since volume conduction (assumed to be instantaneous for simple media) is known to be faster than cable (nerves) conduction we expect that the processing of short EEG windows to identify “respiratory drive onset” at the scalp should provide faster control than current procedures that measure and threshold the signal transmitted through the nerves to the diaphragm.

A more fundamental research challenge concerns the proof of existence and eventual electrophysiological characterization of the feedback related signals postulated here. This assessment will initially require carefully designed ERP experiments in healthy controls exposed to different respiratory loads and during both modes of breathing; voluntary and automatic (e.g., during sleep).

## 3. Conclusions

In this paper we have introduced the concept of a patient ventilator interface (PVI) as a new field of application of the experience gained over last decades in the development of motor oriented brain computer interfaces. As shown here, this clinical application, still at its infancy, is likely to benefit a much larger number of patients than the conventional motor interfaces. On the other hand the transient nature of MV, used mainly during short periods of time or during parts of the day, does not justify the use of permanent interfaces based on invasive methods using intracranial measurements. Thus, we have resorted to a generalization of the non-invasive brain computer interface that opens the way to novel control modalities and new lines of research.

We have here discussed some of the control signals that can be used for the on-line implementation of PVI as well as their advantages and limitations. As novel control signals we have proposed the use of EEG signals containing the instantaneously (volume) conducted respiratory neural drive and the naturally occurring corollary/afferent signals that can help to assess the synchrony between the patient and the machine which we speculate should emerge in the form of feedback related potentials. These questions certainly open an avenue of research that concerns not only the field of BCI but also the electrophysiology of breathing in humans.

As with any emerging technology, the scientific and technological challenges to be addressed are formidable. In particular, the possibility of detecting the neural drive from brainstem generated signals is one of the scientific challenges that might be addressed by a convenient arrangement of the EEG sensors [51] and the exploitation of the spectral selectivity attributed to these signals in the high/very high frequency range. Identifying the existence and electrophysiological characteristics of feedback related potentials to be used for the on-line monitoring of the patient ventilator synchrony is a second scientific aspect to be solved in the near future.

Technological, e.g., better EEG sensors, and signal processing challenges within PVI are similar to those encountered within motor BCI. Consequently, progress within one modality will contribute to progresses in the other. Considering the substantial increase in BCI research nowadays we should expect further progresses in these aspects over a relatively short period of time.
